# Micelle and Inverse Micelle Structure Driven Viscoelasticity and Phase Separation in 2‑Isobutoxyethanol–Water Mixtures: Insights from All-Atom Simulations

**DOI:** 10.1021/prechem.5c00104

**Published:** 2025-12-08

**Authors:** Mayank Dixit, Kenji Sugase, Takashi Taniguchi

**Affiliations:** † Graduate School of Engineering, 12918 Kyoto University, Nishikyo-ku, Kyoto 615-8510, Japan; ‡ Graduate School of Agriculture, Department of Applied Life Sciences Biopolymer Chemistry (concurrently Applied Structural Biology) N346, Faculty of Agriculture Building, 12918 Kyoto University, Kitashirakawa Oiwake-cho, Sakyo-ku, Kyoto 606-8502, Japan

**Keywords:** 2-isobutoxy ethanol, micellar structure, inverse micellar structure, cluster, hydrogen-bond, viscoelasticity

## Abstract

The molecular association between 2-isobutoxyethanol (IBE) and water in mixed polar solvents was investigated by using all-atom molecular dynamics simulations over a wide composition range. Structural analyses, including radial distribution functions (RDFs) and potentials of mean force (PMFs), reveal that hydrogen bonds between IBE-water are significantly stronger than those in IBE–IBE and water–water pairs, as evidenced by higher RDF peak intensities, deeper contact minima, and slower hydrogen-bond autocorrelation decay. These robust interactions promote the formation of large, stable heterogeneous clusters connected via hydrogen-bond networks. Dynamical properties show a pronounced slowdown of IBE self-diffusion with decreasing the mole fraction of 2-isobutoxyethanol (*x*_IBE_). We observed the presence of the inverse micelle structure in *x*_IBE_ = 0.5 and micelle structures in *x*_IBE_ = 0.1. Stress–stress autocorrelation functions indicate enhanced viscoelasticity at *x*_IBE_ ≤ 0.5 due to the presence of inverse micelle and micelle structures, and predominantly liquid-like behavior at *x*_IBE_ > 0.5. At *x*_IBE_ = 0.1, phase separation is observed, consistent with previous experimental research (J. Chem. Thermodynamics, 2000, vol. 32, 729–741). This combined structural–dynamical picture highlights the critical role of hydrogen bonding in controlling microstructure, viscoelastic response, and phase stability in amphiphile–water mixtures, offering insights for designing functional aqueous solutions in industrial and biochemical applications.

## Graphical abstract

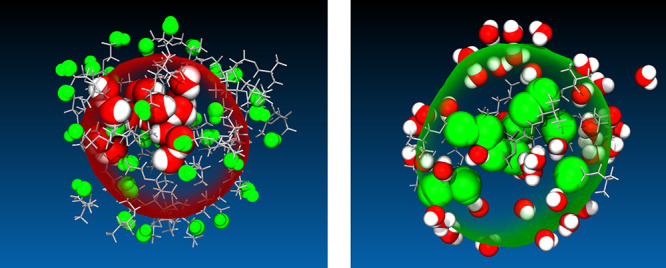

## 1 Introduction

The mixture of 2-isobutoxyethanol (IBE) and water with a low mole fraction of IBE is known to exhibit a lower critical solution temperature (LCST) at 299.15 K, where the solution separates into two liquid phases at a specific temperature.^1–5^ The LCST of binary mixtures plays a vital role in many chemical reactions, in the formation of aggregates of macromolecules of protein and polymers.^4,6,7^ The thermodynamic properties of IBE-water mixtures have been extensively investigated below and above the LCST temperature.^2,3,5,8^ The solvation structure of solvents in binary solvent mixtures is the key factor in determining the critical temperature and compositions of the LCST.^5,9–16^ Perron et al.^2^ measured the thermodynamic properties of IBE-water mixtures and observed the existence of two distinct liquid phases at low IBE mole fractions. The various physical properties and structure of IBE-water mixtures have been measured to investigate the liquid–liquid phase behavior as a function of temperature and mole fraction of IBE.^17–30^ Various experimental studies have reported that the sharp change in the different thermodynamic properties of IBE-water mixtures signifies the liquid–liquid phase separation at room temperature.^17,25–30^ Light scattering experimental measurements reported the presence of large-sized aggregates of isobutoxyethanol in IBE-water mixtures.^31,32,32–34^ Other experimental investigations based on physical properties such as density, enthalpy, diffusion, viscosity, heat capacities, and ultrasound propagation exhibit the presence of heterogeneity in IBE-water mixtures near the lower critical solution temperature.^17,18,20–30^ Previous molecular dynamics simulations have shown that dilute aqueous solutions of 2-butoxyethanol (BE) exhibit hydrophobic tail–driven aggregation at very low concentrations, with aggregate size increasing with temperature and concentration.^11,12^ Previous large-scale molecular dynamics simulations of BE–water mixtures at 298 K^12^ revealed the formation of sizable BE aggregates beginning at a very low mole fraction of BE. Earlier simulation studies have reported difficulties in accurately capturing the liquid–liquid phase separation of BE–water mixtures near the LCST due to artifacts associated with periodic boundary conditions.^11^ Given the chemical similarity between 2-butoxyethanol (BE) and 2-isobutoxyethanol (IBE), such aggregation behavior near the lower critical solution temperature is expected to influence the solvation structure and phase behavior of IBE/water mixtures as well. Consequently, the solvation structure of (2-isobutoxyethanol ((CH_3_)_3_CH–OCH_2_CH_2_–OH)), IBE)-water mixtures remains inadequately understood. In the present work, we investigate the solvation structure and intermolecular associations of IBE–IBE, water–water, and IBE–water pairs across a range of mixture compositions. Special focus was placed on the isobutoxyethanol–water mixture system, which has a lower critical demixing point at room temperature. The details of the system are listed in Table 1.

**Table 1:** Solvent 1 = Isobutoxyethanol (IBE); Solvent 2 = Water (W); *x*_IBE_ = Mole Fraction of IBE; *x*_W_ = Mole Fraction of Water; *n*_IBE_ = Number of Molecules of IBE; *n*_W_ = Number of Molecules of Water; in the Cubic Simulation Cell of Box Length *L*; and ρ is the Density at 299 K^a^

compositions	*x*_IBE_	*x*_W_	*n*_IBE_	*n*_W_	ρ (g/cm^3^)	ρ (g/cm^3^) Exp.^b^	⟨*L*⟩ (nm)
1	1.0	0.0	6000	0	0.885 ± 0.004	0.886	11.1185 ± 0.0002
2	0.9	0.1	5400	600	0.884 ± 0.004	0.889	10.7101 ± 0.0002
3	0.5	0.5	3000	3000	0.903 ± 0.004	0.909	9.1275 ± 0.0002
4	0.1	0.9	600	5400	0.969 ± 0.004	0.961	6.6507 ± 0.0002

a The average box length ⟨*L*⟩ for each composition (NPT ensemble). To perform a comprehensive analysis, the full-atom molecular dynamics simulations were conducted in the following sequence, namely, NPT equilibration (100 ns), NPT production run (3000 ns), and an additional NVT production run (300 ns). The NVT production run is used to calculate the relaxation modulus *G*(*t*), and the NPT production run is used to evaluate equilibrium properties other than *G*(*t*).

b Exp.^5^ at 298.15 K.

## 2 Methods and Computational Details

### 2.1 All-Atom MD Simulation Details

In this study, we conducted all-atom molecular dynamics simulations for equilibrium states of isobutoxyethanol–water mixture systems with different compositions shown in Table 1. We performed all-atom molecular dynamics simulations to study the equilibrium properties of isobutoxyethanol–water mixtures at different compositions, as listed in Table 1. The initial configurations (Figure S1 of the Supporting Information) of all systems were generated by the packmol software^35^ and the multicomponent assembler of CHARMMM-GUI.^36^ These initial configurations were subsequently employed for all-atom molecular dynamics (MD) simulations using GROMACS (version 2019.2).^37^ To generate the necessary input files compatible with GROMACS for each melt system, CHARMM-GUI was employed.^38,39^ The CHARMM general all-atom force field,^40^ was employed for the all–atom MD simulations. The TIP3P model was used for water molecules.^41^ The temperature and pressure were set to 299 K and 1 bar, respectively. In order to attain an equilibrium state for each system (IBE-Water) as outlined in Table 1, the initial configurations underwent an energy minimization process. Subsequently, energy-minimized structures were employed to perform NPT equilibrations (the simulation times for each step are presented in Table 1).

The resulting equilibrated structures were then subjected to a 3000 ns NPT production run (NPT production trajectories were saved every 2 ps). The generated trajectories were utilized to compute the dipole–dipole autocorrelation function, radial distribution function, and potentials of mean force, hydrogen-bond (H-bond) autocorrelation, and self-diffusion coefficients of IBE and water molecules in IBE-water mixtures. These calculations facilitated the elucidation of the formation of homo- and heterogeneous clusters between IBE and water molecules. We additionally performed 300 ns of NVT production for each system to compute stress–stress autocorrelation functions. To control the system temperature, the Nosé-Hoover thermostat^42,43^ was employed with a coupling constant of 1 ps. The pressure was maintained at 1 bar during both equilibration and production runs using the Berendsen barostat and Parrinello–Rahman barostat.^44^ For equilibration, a time constant of 5 ps and a compressibility of 4.5 × 10^–5^ bar^–1^ (4.5 × 10^–10^ Pa^–1^) were employed. The Verlet cutoff scheme with a cutoff radius of 1.2 nm was utilized to construct the neighbor list in all-atom MD simulations. Hydrogen bond lengths were constrained using the LINCS algorithm.^45^ A time step of 2 fs was employed for the all-atom molecular dynamics simulations. The particle mesh Ewald method^46^ was employed to calculate the electrostatic interactions with a real-space cutoff of 1.2 nm, Fourier spacing of 0.12 nm, and a relative accuracy of 1.0 × 10^–5^. The estimated values of densities of these IBE-water mixtures are in good agreement with the reported experimental densities,^5^ as shown in Table 1.

## 3 Results and Discussion

### 3.1 Association of Isobutoxyethanol and Water

The present section is dedicated to exploring the associations between the IBE and water molecules in the IBE-Water mixtures. We present snapshots of the IBE-Water mixtures obtained after a 3 μs production run in Figure 1.

**Figure 1:**
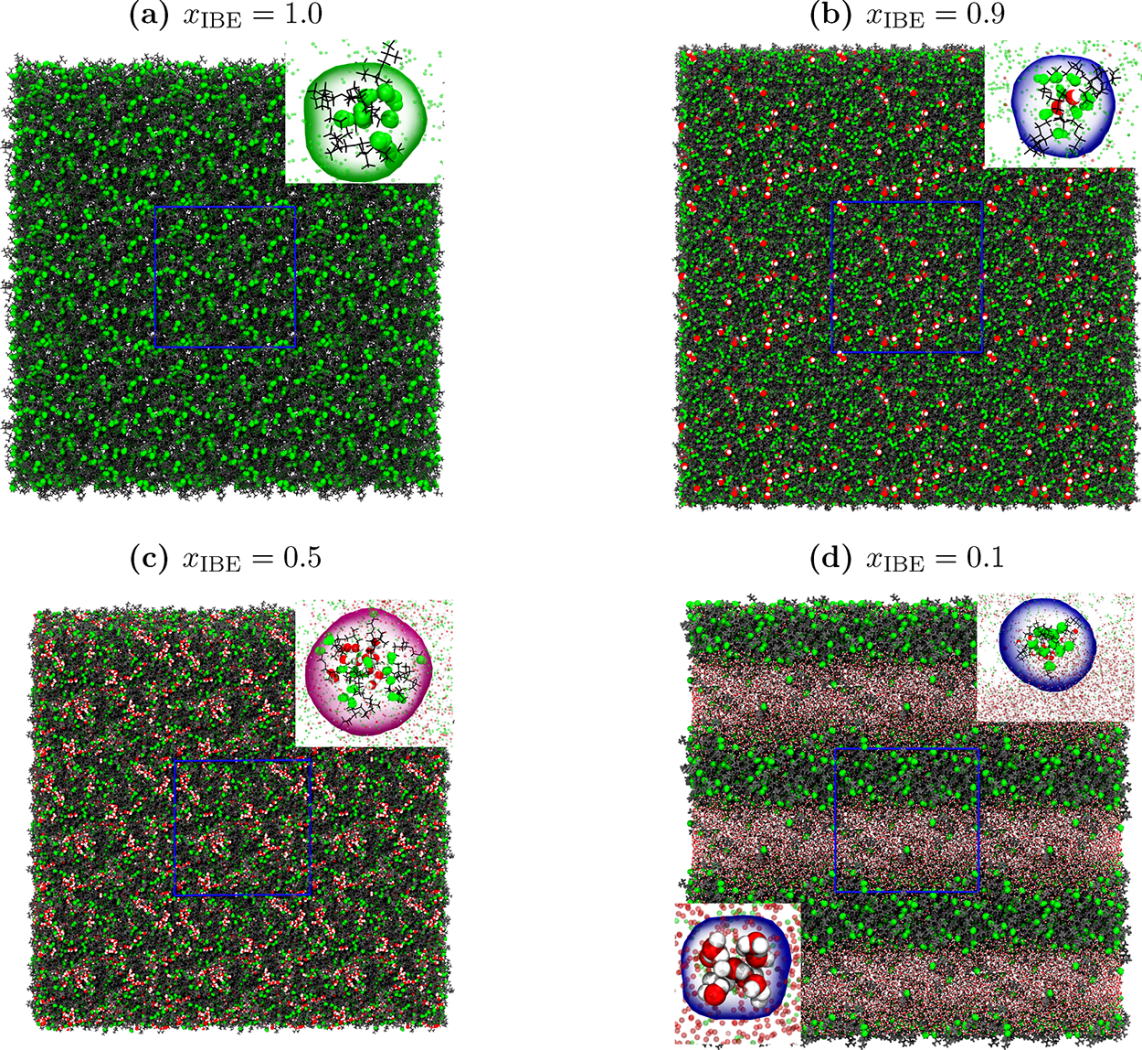
Snapshots of mixtures of IBE-Water, i.e., *x*_IBE_ = 1.0 (a), *x*_IBE_ = 0.9 (b), *x*_IBE_ = 0.5 (c), *x*_IBE_ = 0.1 (d) in equilibrium. The hydrogen and oxygen atoms of water are colored white and red colors, respectively. The hydroxy group of IBE is shown in green, and the tail is shown in gray color. We have also shown the zoomed-out of these figures in the inset. The insets of figures (a)–(d) respectively represent the small size IBE cluster (inset of figure (a)), small size water cluster is surrounded by IBE molecules (inset of figure (b)), large size water cluster is surrounded by IBE molecules (inset of figure (c)), and large size IBE cluster is surrounded by water molecules (right top inset of figure (d)), and the large size water cluster (bottom left inset of figure (d)). The simulation box is shown by the blue color frame in each figure.

In Figure 1, we identified two distinct types of heterogeneous clusters in IBE–water mixtures. The first type consists of IBE molecules forming a hydrogen-bonded network that encloses water molecules, resembling an inverse micelle (Type-I). The second type comprises hydrophobically aggregated IBE clusters, which are surrounded by water molecules hydrogen-bonded to alcoholic OH groups, akin to conventional micelles (Type-II). The previous experimental studies have reported these two types of clusters.^5,18,47^ Type-I clusters were observed for *x*_IBE_ > 0.1, and type-II clusters were observed for *x*_1_ ≤ 0.1. At *x*_IBE_ = 0.5, IBE molecules predominantly form inverse micelle-like clusters encapsulating water, consistent with previous reports.^5,18,47^ At *x*_IBE_ = 0.1, we observed clear liquid–liquid phase separation, in agreement with earlier experimental observations.^5,18,47,48^

To explore the association of IBE-IBE, IBE-water, and water–water molecules, an analysis was conducted by calculating the radial distribution functions (RDFs) between IBE and water. The IBE-IBE, IBE-water, and water–water radial distribution function is defined as the ratio of the local density of the component site at distance *r* from another component site and the bulk component density. The radial distribution functions *g*_αβ_(*r*) are defined by the following equation:

$$g_{\alpha \beta }(r)=\frac{\langle \rho_{\beta }(r)\rangle_{\mathrm{local},\alpha }}{\langle \rho_{\beta }(r_{c})\rangle_{\alpha }}$$ 1

Herein, ⟨ρ_β_(*r*)⟩_local,α_ represents the local mean particle density of β particles in the vicinity of α particles, measured at radial distance *r*. The denominator within the expression on the right-hand side of eq 1, denoted as ⟨ρ_β_(*r*_c_)⟩_α_, signifies the mean density of particles of type β enclosed within a spherical volume of radius *r*_c_, centered at the location of the α particle. The value of *r*_c_ is specifically established as half of the simulation box’s dimensions. In our investigation, we explored the radial distribution functions of specific molecular interactions in the IBE-Water mixtures, which are shown in Figure 2. Specifically, we estimated the RDFs of [[O_(OH)_]_IBE_] around [[H_(OH)_]_IBE_] (representing the RDF of the oxygen atom of the hydroxy group of IBE molecules around the hydrogen atom of the hydroxy group of another IBE molecules), [[O_(OH)_]_IBE_] around [[O_(OH)_]_IBE_] (representing the RDF of the oxygen atom of the hydroxy group of IBE molecules around the oxygen atom of the hydroxy group of another IBE molecules), and [[H_(OH)_]_Water_] around [[O_(OH)_]_IBE_] (representing the RDF of the oxygen atom of the hydroxy group of IBE molecules around the hydrogen atom of the hydroxy group of water molecules). Furthermore, we examined the RDFs of [[O_(OH)_]_water_] – [[H_(OH)_]_water_] (representing the hydrogen atom of hydroxy groups of water molecules around the oxygen atom in another OH group of another water molecules) and [[O_(OH)_]_water_] – [[O_(OH)_]_water_] (representing the oxygen atom of hydroxy groups of water molecules around oxygen atom of hydroxy groups of another water molecules. Notably, we observed a distinctive peak at a distance of 0.2 nm in the [[O_(OH)_]_water_] – [[H_(OH)_]_IBE_] RDF, indicating a strong association between IBE and water molecules. The local density of [[O_(OH)_]_IBE_] surrounding [[H_(OH)_]_Water_] exhibits a higher value compared to the local densities of [[O_(OH)_]_water_] surrounding [[H_(OH)_]_Water_]. The association of [[O_(OH)_]_water_] – [[H_(OH)_]_IBE_] is facilitated by hydrogen bonding, providing compelling evidence for the establishment of heterogeneous clusters between IBE and the water molecules. We have shown the RDFs between the backbone of IBE molecules in Figure S2 of the Supporting Information. We found that as the mole fraction of IBE changes from 1.0 to 0.5, there is a marginal change in the RDFs peak intensity, while changing the mole fraction from 0.5 to 0.1, the first RDF peak intensity is significantly enhanced, which confirms the strong hydrophobic association between the backbone of IBE molecules in the IBE-water mixture with *x*_IBE_ = 0.1. The presence of a highly packed hydrophobic backbone of IBE molecules supports the formation of micellar structures in the mixture with an *x*_IBE_ = 0.1.

**Figure 2:**
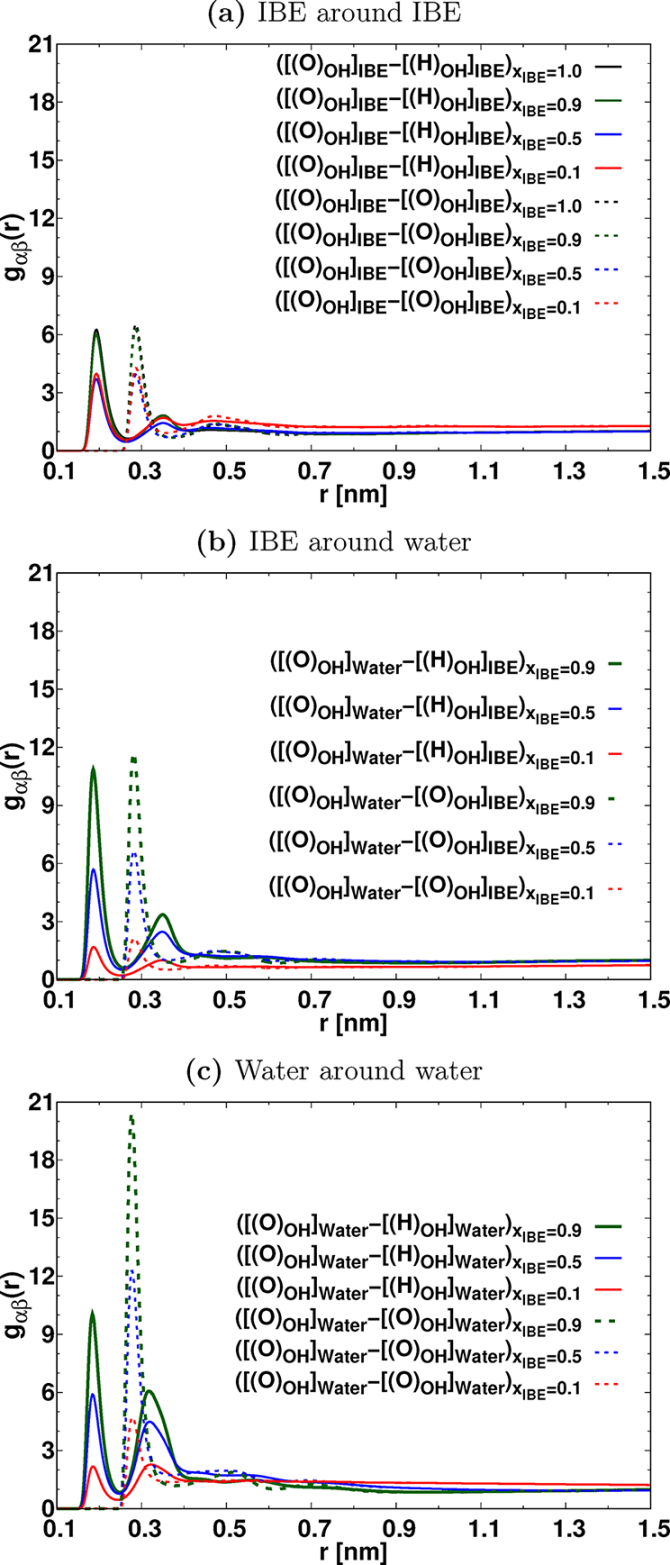
Radial distribution functions (RDFs) between IBE-IBE (a), IBE-water (b), and water–water (c) in the IBE-water mixtures.

We have investigated the solvation structure of the IBE around water, IBE around IBE, and water around water. To achieve this, we employ eq 2 to compute the running coordination numbers (RCNs) delineating the spatial arrangement of IBE around water, water around water, and IBE around IBE. The coordination number is defined as

$${\mathrm{RCN}}_{\alpha \beta }(r)=4\pi \rho_{\beta }\int_{r_{0}}^{r}(r^{\prime }{)}^{2}g_{\alpha \beta }(r^{\prime })dr^{\prime }$$ 2

In this context, RCN_αβ_(*r*) denotes the number of type β atoms surrounding species α, confined within a radial shell extending from *r*_0_ to *r*. Here, ρ_β_ represents the number density of β in the system, while *g*_αβ_(*r*) stands for the radial distribution function. The latter provides the ratio of the local density of β around α to the bulk density of β. For the calculation of the first solvation shell coordination number, *r*_0_ is set to zero. The running coordination numbers RCN_αβ_(*r*) are depicted in Figure 3.

**Figure 3:**
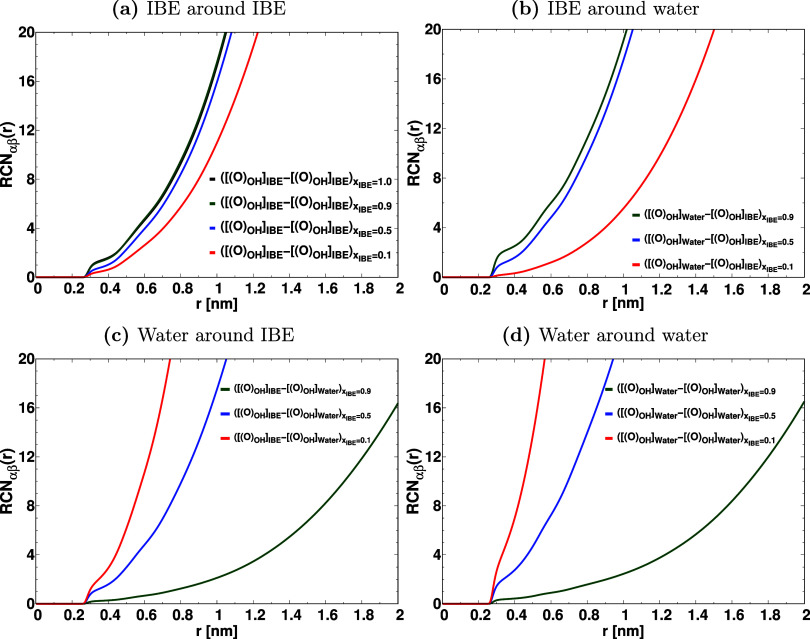
Running coordination numbers RCN_αβ_(*r*) of (a) IBE around IBE, (b) IBE around water, (c) water around IBE, and (d) water around water molecules in the mixture. “[[(O)_OH_]_IBE_]” represents the oxygen atom in the isobutoxy ethanol, and [[(O)_OH_]_Water_] stands for the oxygen atom of the hydroxy group of water. The RCNs are calculated by considering [[(O)_OH_]_Water_], [[(O)_OH_]_IBE_] as α and β particles in the expression *g*_αβ_(*r*).

From the analysis of Figure 3 and Table S1 of the Supporting Information, it is evident that the population of IBE molecules around IBE is markedly decreased as the mole fraction of IBE is changed from *x*_IBE_ = 0.9 to *x*_IBE_ = 0.1. In the case of *x*_IBE_ = 0.1, 0.5, the running coordination numbers of water around water are significantly larger in comparison to the mixtures with *x*_IBE_ = 0.9. Therefore, the large-sized water clusters are formed in the mixtures with *x*_IBE_ = 0.1, 0.5. These large-sized water clusters in the mixture with *x*_IBE_ = 0.5 are surrounded by IBE molecules (as shown in Figure 3b), which support the formation of inverse micelle structures (Type-I) as shown in Figure 1c. The previous experimental research also reported the formation of inverse micelle structures.^5,18,47,48^ In the case of *x*_IBE_ = 0.1 mixture (as shown in Figure 3c), the running coordination number of water around IBE is substantially larger than that of the mixture with *x*_IBE_ = 0.9, which suggests the presence of micromicellar structure as shown in Figure 1d. From the analysis of Table S1, it is seen that the number of IBE around water in the first and second coordination shells is substantially higher in mixture with *x*_1_ = 0.9 and *x*_1_ = 0.5 than that of *x*_1_ = 0.1, while the number of water around IBE is significantly larger in *x*_1_ = 0.1 in comparison to *x*_1_ = 0.9 and *x*_1_ = 0.5 mixtures. The number of water molecules around water in the first and second coordination shells is higher in *x*_1_ = 0.5 than that of *x*_1_ = 0.9, which confirms the formation of a small-sized water cluster surrounded by IBE in *x*_1_ = 0.9, and a large-sized water cluster surrounded by IBE in *x*_1_ = 0.5. Therefore, we can confirm the formation of inverse micelle in *x*_1_ = 0.9 and *x*_1_ = 0.5 and direct micellar structure in *x*_1_ = 0.1 mixtures.

The potentials of mean force (PMFs) are widely utilized to investigate the stability of clusters, as demonstrated in various studies.^49–62^ Accordingly, in this study, we have computed the potentials of mean force between the IBE and water molecules, employing the following equation:

$$W(r)=-k_{B}T\log(g(r))$$ 3

Here, *k*_B_ signifies the molar gas constant (in kJmol^–1^/K), *T* denotes the system’s temperature, and *g*(*r*) represents the radial distribution function between IBE and water molecules. Figure 4 illustrates the potentials of mean force between the two IBE-IBE, Water–Water, and IBE-Water as functions of their distance.

**Figure 4:**
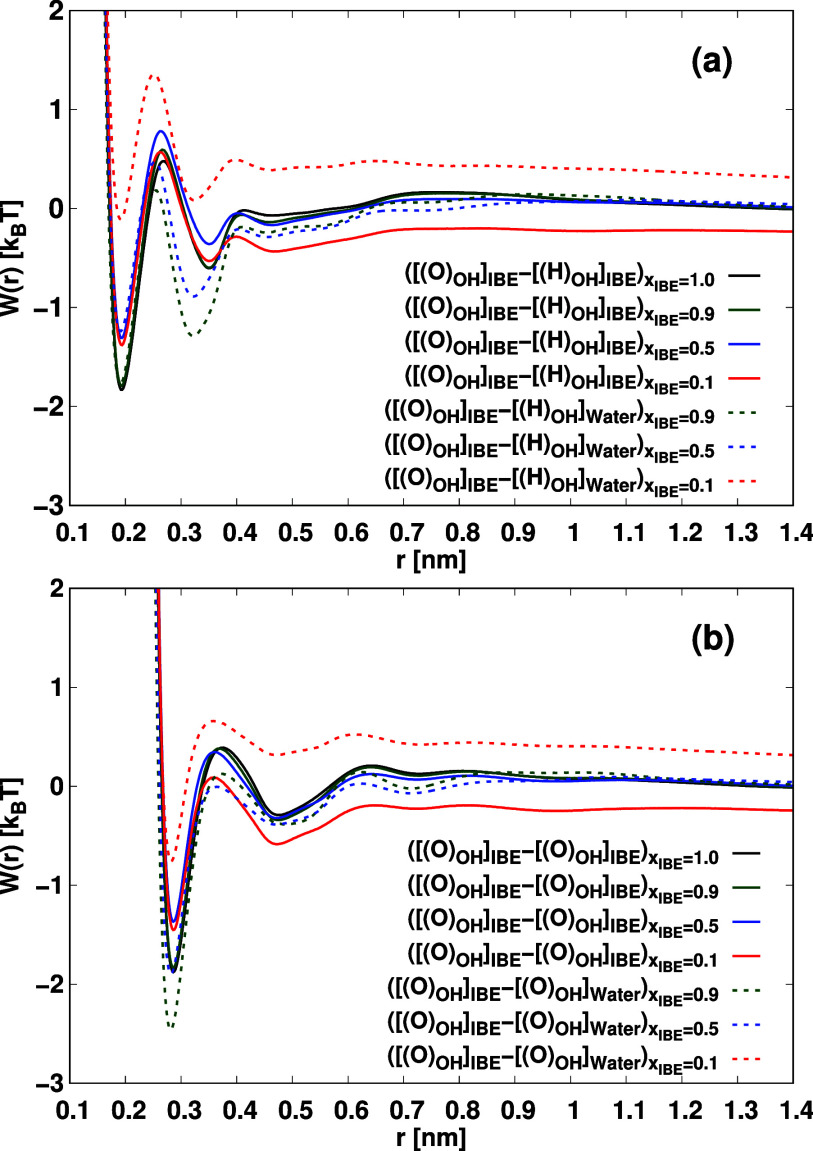
Potentials of mean force (*W*(*r*)) of [[O_OH_]_IBE_] – [[H_OH_]_IBE_] and [[O_OH_]_IBE_] – [[H_OH_]_water_] (a) and [[O_OH_]_IBE_] – [[O_OH_]_IBE_] and [[O_OH_]_IBE_] – [[O_OH_]_water_] (b). Potentials of mean force (*W*(*r*)) is computed by using the equation *W*(*r*) = – *k*_B_ *T*log­(*g*(*r*)). Here, *k*_B_ represents the molar gas constant in units of kJmol^–1^/K, *T* denotes the system temperature, and *g*(*r*) corresponds to the radial distribution function between the hydroxy groups of IBE and water. The magnitude of error bars in each PMF profile is less than 0.5 *k*_B_ *T*.

We evaluated the interaction free energy between IBE-IBE, IBE-water, and water–water pairs using the potentials of mean force *W*(*r*) (Figure 4). As seen in Figure 4a, the interactions [[O_OH_]_IBE_] – [[H_OH_]_IBE_], [[O_OH_]_water_] – [[H_OH_]_water_], and [[O_OH_]_IBE_] – [[H_OH_]_water_] exhibit a sharp contact minimum around 0.2 nm, with free energy depths significantly exceeding thermal energy. These results indicate that the association between [[O_OH_]_water_] – [[H_OH_]_IBE_] is more stable than that of [[O_OH_]_IBE_] – [[H_OH_]_IBE_] and [[O_OH_]_water_] – [[H_OH_]_water_]. Additionally, we identified contact minima, solvent-shared minima (SShM), and solvent-separated minima (SSM) for [[O_OH_]_water_] – [[H_OH_]_IBE_], [[O_OH_]_IBE_] – [[H_OH_]_IBE_], and [[O_OH_]_water_] – [[H_OH_]_water_]. [[O_OH_]_water_] – [[O_OH_]_IBE_]. Utilizing the PMF results, we establish a criterion for determining stable associations between two molecules. A stable association is deemed to occur when the PMF (*W*) between two molecules satisfies the inequality: (|*W*(*r*_CM_)| should be sufficiently larger than the thermal energy, with *r*_CM_ representing the distance at the contact minimum (CM)). Such stable associations are capable of forming a “cluster.” To assess the number of molecules participating in a cluster, we apply two criteria: (i) the distance criterion (*r*_αβ_ < 0.6 nm) and (ii) the criterion for stable association (|*W*(*r*_CM_)| > *k*_B_ *T*), where *r*_αβ_ is the distance between molecular site α and β. α and β stand for molecular sites (α, β ∈{[[(O)_OH_]_IBE_], [[(O)_OH_]_Water_]}). The resulting number of clusters with a size of *s* is denoted as *n*_*s*_^(αβ)^(*k*;|*W*(*r*_CM_)| > *k*_B_ *T*). The notation adopted to distinguish different types of clustering among hydroxy groups of IBE and water is defined as follows. When a cluster consists exclusively of a single type of hydroxy group, denoted as α, the notation *n*_*s*_^αα^ represents homogeneous clusters. Conversely, if the cluster comprises hydroxy groups originating from different types of molecules, α and β, it is denoted by *n*_*s*_^αβ^, which represents heterogeneous clusters. The formation of inverse micelles and micelle structures can be recognized by homo- and heterogeneous cluster analysis. Therefore, we perform homogeneous and heterogeneous cluster analyses to investigate the formation of micelle and inverse micelle structures. As a notation for homogeneous clusters, we will use *n*_*s*_^(X)^ instead of *n*_*s*_^(αα)^, where X is “IBE” when α is [[(O)_OH_]_IBE_], and is “Water” when α is [[(O)_OH_]_Water_]. Namely, *n*_*s*_^(IBE)^ corresponds to clusters of size *s* consisting solely of IBE molecules, and *n*_*s*_^(Water)^ represents homogeneous clusters of water molecules. For heterogeneous clusters, we use the notation *n*_*s*_^((IBE)(Water))^, which represents the heterogeneous clusters of the IBE and water molecules. Furthermore, to express a size *s* heterogeneous cluster symbolically, we use the symbol (IBE)_*p*_(Water)_*q*_, which means the cluster is composed of *p* IBE and *q* water molecules (*s* = *p* + *q*).

As shown in Figure 6c,d, we can expect formations of two types of heterogeneous clusters, *p* ≪ *q* and *p* ≫ *q*. To know which type of heterogeneous clusters is dominantly existing in each system, we focused on two representative heterogeneous cluster cases specified by (*p* = 1, *q* > 1) and (*p* > 1, *q* = 1). (*p* = 1, *q* > 1) means clusters composed of one IBE molecule and *q* water molecules, on the other hand, (*p* > 1, *q* = 1) means a cluster composed of one water molecule and *p* IBE molecules. Namely, we analyzed the size distribution of two distinct types of heterogeneous clusters *n*_*s*_^((IBE)*p* (Water)*q*)^ with *p* = 1 and *q* > *p*, corresponding to inverse micellar structures, in which a cluster of water molecules is surrounded by a single IBE molecule. The second type, characterized by *p* > *q* with *q* = 1, represents micromicellar structures where a cluster of IBE molecules is encapsulated with a single water molecule. Furthermore, the trends observed in these simple heterogeneous cluster motifs are consistent with more complex cluster types, namely, *n*_*s*_^((IBE)*p* (Water)*q*)^ for *q* > *p* and for *p* > *q*, suggesting a generalizable framework for understanding the inverse micromicellar cluster and micromicellar cluster structures in IBE-water mixtures.

To quantify the number of clusters of a given size, *s*, we analyze *K* configurations sampled from the final 1000 ns of the 3000 ns simulation trajectories. Here, *K* denotes the number of frames extracted within the 1000 ns sampling window, such that *n*_*s*_^(X)^(*k*) (where X is IBE, water, and (IBE)_p_(Water)_q_ with *p* + *q* = *s*) is evaluated for *k* = 1, ···, *K* = 50 000. Using these values, the fraction of cluster formation *f*_cluster_^(X)^(*s*) of size *s* involving hydroxy groups of IBE or water molecules is defined as^51,63–65^

$$f_{\mathrm{cluster}}^{\left(X\right)}(s)=\frac{\frac{1}{K}\sum_{k=1}^{K}n_{s}^{\left(X\right)}(k;|W(r_{\mathrm{CM}})|>k_{B}T)}{\left[\sum_{\begin{array}{l} X=(\mathrm{IBE}{)}_{s},(\mathrm{Water}{)}_{s},\\ ((\mathrm{IBE}{)}_{p}(\mathrm{water}{)}_{q},p=1,q>p),\\ \left((\mathrm{IBE}{)}_{p}(\mathrm{water}{)}_{q},q=1,p>q\right) \end{array}}\sum_{s=1}^{\infty }\frac{1}{K}\sum_{k=1}^{K}n_{s}^{\left(X\right)}(k;|W(r_{\mathrm{CM}})|>k_{B}T)\right]}$$ 4

From eq 4, ∑_X_∑_*s* = 1_^*∞*^ *f*_cluster_^(*X*)^(*s*) = 1 holds.

In Figure 5, the cluster-formation-fractions of (a) IBE-cluster, (b) water-cluster, and (c,d) IBE-water mixed cluster are depicted. For the IBE-Water mixture with *x*_IBE_ = 0.1, large size clusters composed only of IBE molecules are observed, ranging in size from 2 to 31 (the schematic diagram is shown in Figure 6a), which supports the formation of micellar structures as seen in Figure 5. In the case of *x*_IBE_ = 0.5, we also noticed the formation of large clusters composed only of water molecules, with sizes ranging from 2 to 31. The schematic diagram of the water cluster is shown in Figure 6b. In Figure 5c, we show the formation of heterogeneous clusters between the IBE and water. In the mixtures with *x*_IBE_ = 0.5 and 0.9, we see the formation of water clusters surrounded by IBE molecules. The schematic diagrams of small-sized water clusters surrounded by IBE molecules and large-sized water clusters surrounded by IBE molecules are shown in Figure 6c, which shows the formation of inverse micromicellar structures as shown in Figure 1. The analysis of running coordination numbers of IBE around water and water around water (Figure 3b,d) also supports the formation of an inverse micromicellar structure in *x*_IBE_ = 0.5. The light green high peaks at *s* = 7 and 8 show that the small-sized water clusters are surrounded by IBE molecules. In addition, larger clusters (*s* ∼ 15) but with very weak peaks are also appearing. In the mixture with *x*_IBE_ = 0.1, we found the existence of heterogeneous clusters (IBE)_p_(Water)_*q*_ (*p* = 3–31 and *q* = 1) (Figure 5d) where large-sized IBE clusters are enveloped by water molecules, which supports the formation of micromicellar structure. The schematic diagram of the micromicellar structure is shown in Figure 6d.

**Figure 5:**
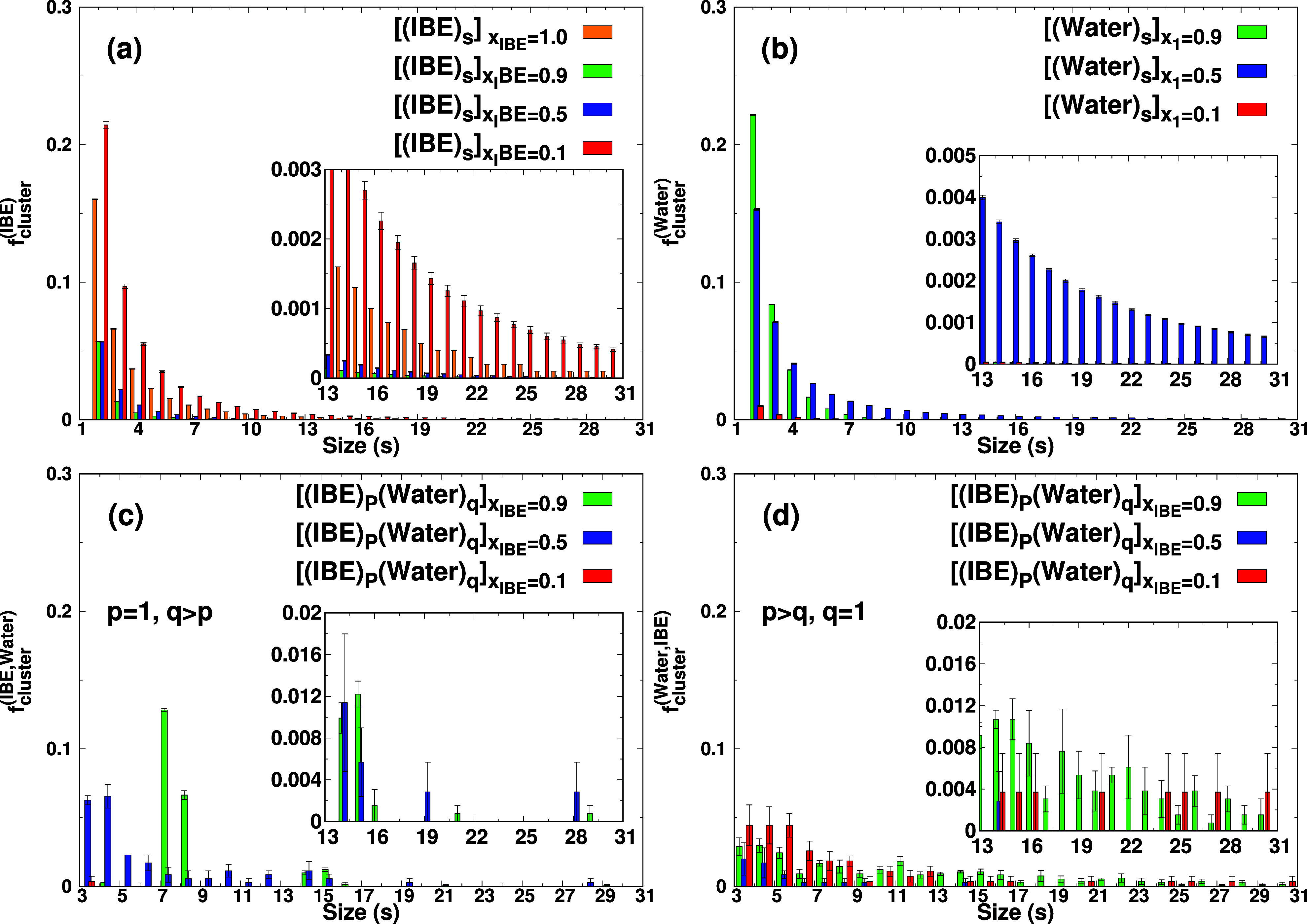
(a) Fraction of size-*s* cluster composed only of IBE *f*_cluster_^(IBE)^(*s*), (b) that only of Water *f*_cluster_^(Water)^(*s*) and (c) that containing both IBE and water *f*_cluster_^((IBE)p(Water)q)^(*s*) with the condition (*p* = 1, *q* > *p*) (d) that containing both water and IBE *f*_cluster_^((Water)*p* (IBE)*q*^)­(*s*) with (*q* = 1, *p* > *q*) in IBE-Water mixtures and the IBE pure system. The fractions for *s* = 1 are not displayed in the figure.

**Figure 6:**
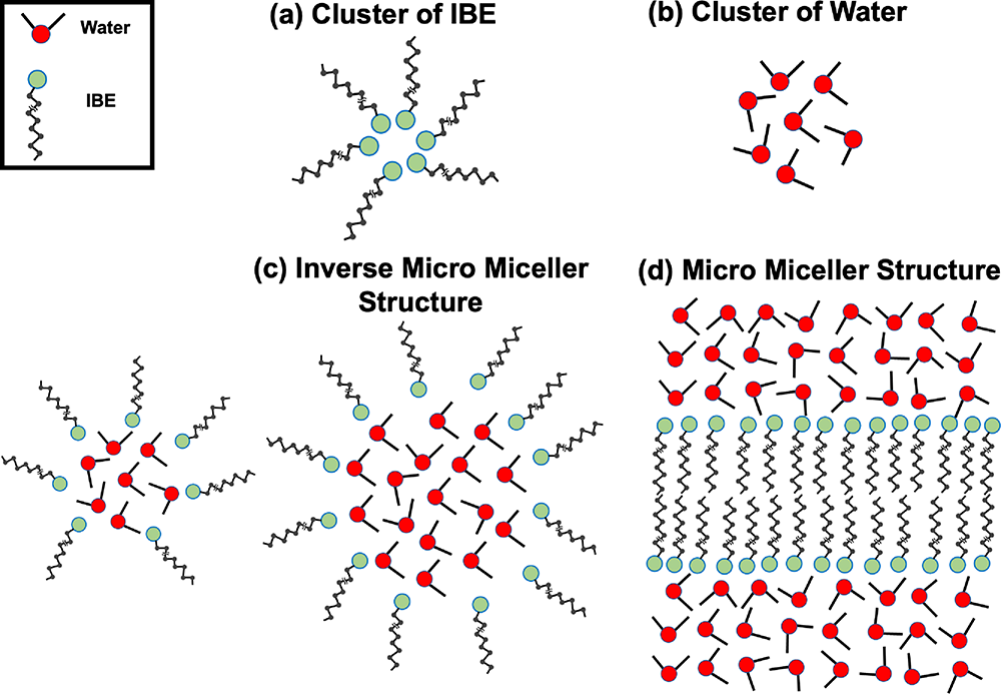
Schematic diagram of different types of cluster structures: (a) IBE cluster, (b) water cluster, (c) inverse micro micellar cluster structure, and (d) micro micellar cluster structure.

### 3.2 Dipole–Dipole Autocorrelation of IBE in IBE-Water Mixtures

We have also estimated the dipole moment autocorrelation function ϕ­(*t*) for each melt system by using the following equation:

$$\phi (t)=\frac{\left\langle \mu (t)\cdot \mu (0)\right\rangle }{\left\langle \mu (0)\cdot \mu (0)\right\rangle }$$ 5

where the vectors **μ**(0) and **μ**(*t*) represent the dipole moment of a single molecule at time *t* = 0 and time *t*, respectively, while the angle bracket denotes an ensemble average. We have illustrated the dipole moment autocorrelation function, denoted as ϕ­(*t*), in Figure 7. We determined the dipole relaxation time by fitting the dipole–dipole autocorrelation function to both a simple exponential function and a stretched exponential function,^66^ defined as follows:

$$\phi (t)=\exp[-(\frac{t}{\tau_{d}}{)}^{\beta_{d}}]$$ 6

The dipole relaxation times, denoted as τ_d_, and the associated stretching exponents, represented as β_d_. The dipole–dipole autocorrelation function ϕ­(*t*) is also fitted using the Kohlrausch–Williams–Watts stretched exponential function to determine the dipole relaxation time τ_d_ and the stretching exponent β_d_, and the values are shown in the Table of Figure 7. A similar trend is observed from the rotational correlation functions (Figure S3 of the Supporting Information). From the analysis of Figure 7b, we can see that the average relaxation times τ_d_ obtained by using β = 1 and β < 1 are similar, although the dipole relaxation times estimated by using stretched exponential functions are always respectively smaller than the ones by using simple exponential decay functions. The dipole–dipole relaxation of IBE at *x*_IBE_ = 0.9 is markedly slower than that at *x*_IBE_ = 1.0, indicating that even a small fraction of water can significantly hinder IBE dynamics. At *x*_1_ = 0.5, the relaxation remains slower than in the pure system (*x*_IBE_ = 1.0), though slightly faster than at *x*_IBE_ = 0.9. At *x*_IBE_ = 0.1, the relaxation dynamics again become slower. Namely, we observed two types of behaviors of ϕ­(*t*), (i) the faster relaxation of ϕ­(*t*) for (a) *x*_IBE_ = 1.0 and (d) *x*_IBE_ = 0.1, and (ii) the slower relaxation for (b) *x*_IBE_ = 0.5 and (c) *x*_IBE_ = 0.9. From the snapshots of the system shown in Figure 1, the reason we observed the two different behaviors can be understood. In (a), *x*_IBE_ = 1.0, an IBE is surrounded by other IBEs, and the dynamics of IBE molecules are determined by interactions from the surrounding IBEs. In *x*_IBE_ = 0.1, the system shows a phase separation (Figure 1d), and the dynamics of an IBE in the IBE-rich phase can be similar to those in (a), although there is a relatively smaller contribution to ϕ­(*t*) from the dipole moment of IBEs located at an interface between phase-separated phases. In (b) *x*_IBE_ = 0.9 and (c) *x*_IBE_ = 0.5, large-sized water clusters are present (Figure 5), while small-sized water clusters are identified in *x*_IBE_ = 0.1. As seen in the analysis of the potential of the mean force *W*(*r*) among IBE and water molecules, the attractive interactions between IBE and water and IBE and IBE in systems (b) and (c) are stronger than those in systems (a) and (d). Therefore, the dynamics of IBE in (b) and (c) systems are slowed down compared with those in (a) and (d).

**Figure 7:**
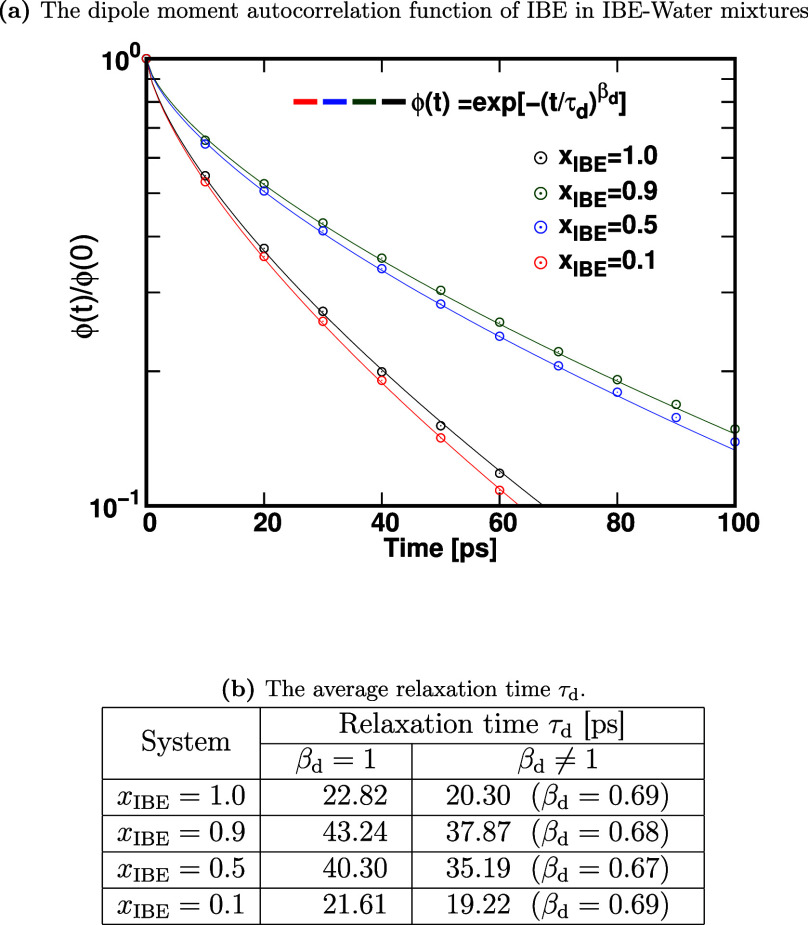
(a) Dipole moment autocorrelation function of IBE in IBE-water mixtures where the fitted lines are shown using β ≠ 1. (b) Table of the dipole relaxation times τ_d_ for the four *x*_IBE_ mixtures. The dipole moment autocorrelation function ϕ­(*t*) is fitted using exponential functions with β_d_ = 1 or β_d_ ≠ 1 to determine relaxation time τ_d_.

### 3.3 Self-Diffusion Coefficient of IBE and Water in IBE-Water mixtures

This section focuses on the exploration of the respective self-diffusion behavior of IBE and water molecules in the mixture. To assess the mean square displacement, the final 1000 ns of the 1500 ns trajectories in the NPT production runs were considered. Figure 8 presents the time evolution of the mean square displacement (MSD) of the center of mass for the IBE and water. The self-diffusion coefficients *D* of IBE and water molecules were quantified by employing the Einstein relation, defined as follows.

$$D=\lim_{t\to \infty }\;\frac{\left\langle (R_{\mathrm{cm}}(t)-R_{\mathrm{cm}}(0){)}^{2}\right\rangle }{6t}$$ 7

The mean square displacement of the center of mass of IBE or water molecule, denoted as ⟨(***R***_cm_(*t*) – ***R***_cm_(0))^2^⟩, was calculated, and the resulting self-diffusion coefficients are presented in Table 2. In the presence of a low mole fraction of water in the IBE-Water mixture, a substantial reduction in the motility of IBE was observed. Notably, the introduction of water resulted in the formation of hydrogen bonds between the IBE and water, leading to a substantial decline in the mobility of IBE molecules. The tendency of the change in mobility of IBE molecules by *x*_IBE_ is similar to that of the dipole–dipole autocorrelation function shown in Figure 7 and can be understood in the way explained in the previous subsection.

**Figure 8:**
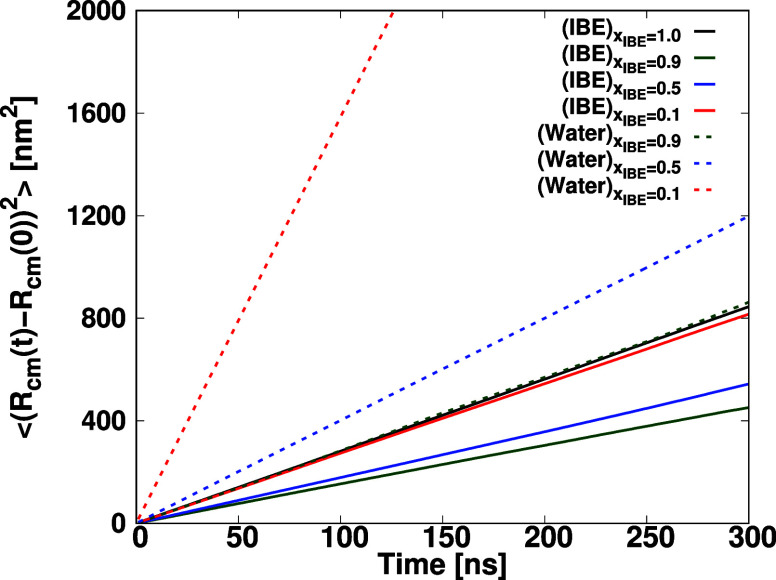
Mean square displacement (MSD) vs time for the centers of mass of IBE and water in the mixtures.

**Table 2:** Self-Diffusion Coefficient *D* of IBE and Water in the IBE-Water Mixture

	self-diffusion coefficient *D* × 10^6^(cm^2^/s)	
fraction of IBE	IBE	Water
*x*_IBE_ = 1.0	4.70 ± 0.02	
*x*_IBE_ = 0.9	2.51 ± 0.06	4.77 ± 0.01
*x*_IBE_ = 0.5	3.08 ± 0.01	6.66 ± 0.02
*x*_IBE_ = 0.1	4.47 ± 0.02	26.47 ± 0.50

### 3.4 Dynamic Behavior of H-Bonds Using IBE and Water in the Mixture

In this section, our focus lies on the exploration of the hydrogen bond dynamics between IBE and water molecules. To achieve this, we calculate the hydrogen bond (HB) time correlation function *C*_HB_(*t*) for pairs *i*, *j* of hydrogen-bonded molecules. This correlation function’s definition is based on the previous seminal works.^67,68^

$$C_{\mathrm{HB}}^{x}(t)=\left\langle \frac{\sum h_{ij}(t_{0})h_{ij}(t_{0}+t)}{\sum (h_{\mathrm{ij}}(t_{0}){)}^{2}}\right\rangle$$ 8

where *h*_*ij*_(*t*) serves as an indicator of whether a pair *i*, *j* satisfies the geometric criteria for hydrogen bonding at time *t*. Specifically, *h*_*ij*_(*t*) = 1 indicates the presence of a hydrogen bond, while *h*_*ij*_(*t*) = 0 signifies its absence. Hydrogen bonds were defined geometrically by a donor–hydrogen–acceptor (D–H···A) angle greater than 130.0° and a donor–acceptor distance shorter than 0.30 nm. The summation is carried out over all possible hydrogen-bonded pairs *i*, *j* of IBE and water molecules, and angular brackets represent an average over different starting times *t*_0_ in the trajectory. In this investigation, we explore the dynamics of hydrogen bonds formed between IBE and water molecules in IBE-Water mixtures. To assess hydrogen bond dynamics, we calculate the hydrogen bond (HB) time correlation function *C*_HB_(*t*) for pairs *i*, *j* of IBE and water. The superscript x distinguishes two different definitions for measuring *h*_*ij*_(*t*) at future points in time: continuous *C*_HB_^c^(*t*) and intermittent *C*_HB_^I^(*t*). These correlation functions offer distinct insights into hydrogen bond dynamics. Continuous hydrogen bond correlation *C*_HB_^c^(*t*) reflects the average time a pair remains intact as a hydrogen bond before breaking, yielding continuous lifetime τ_HB_^c^. In contrast, intermittent hydrogen bond correlation *C*_HB_^I^(*t*) investigates the persistence probability of a hydrogen bond created at *t* = 0, despite multiple breakings and reformations during the time period [0, *t*]. The corresponding lifetime is known as the intermittent lifetime or hydrogen bond relaxation time, τ_HB_^I^. Using the MDAnalysis package,^69^ we estimated the continuous *C*_HB_^c^(*t*) and intermittent *C*_HB_^I^(*t*) hydrogen bond correlation functions for specific pairs, such as [[OH]_IBE_], [[OH]_Water_], as displayed in Figure 9. Moreover, we determined the hydrogen bond relaxation time τ_HB_ by fitting the hydrogen bond autocorrelation functions *C*_HB_^c^(*t*) and *C*_HB_^I^(*t*) into the Kohlrausch–Williams–Watts (KWW) stretched exponential function. The values of τ_HB_ and stretching exponent β_HB_ are presented in Table 3. The intermittent hydrogen bond autocorrelation function was fitted into two types of KWW stretched exponential functions, namely *C*_HB_^*′*I^(*t*) = *C*_HB_^*′*I^(0)­exp­[−(*t*/τ_HB_^*′*I^)^β_HB_^*′*I^^] and *C*_HB_^I^(*t*) = *P*_HB_^I^ + *C*_HB_^I^(0)­exp­[−(*t*/τ_HB_^I^)^β_HB_^I^^]. The fitted functions for the IBE-water mixture with *x*_IBE_ = 0.9 are shown in Figure S4, and χ^2^ values are provided in Table S2 (Table S2 is given in the Supporting Information). Based on the χ^2^ values, *C*_HB_^I^(*t*) yields a better fit compared to *C*_HB_^*′*I^(*t*). The estimated values of τ_HB_^I^, stretching exponent β_HB_^I^, and *P*_HB_^I^ are presented in Table 3.

**Figure 9:**
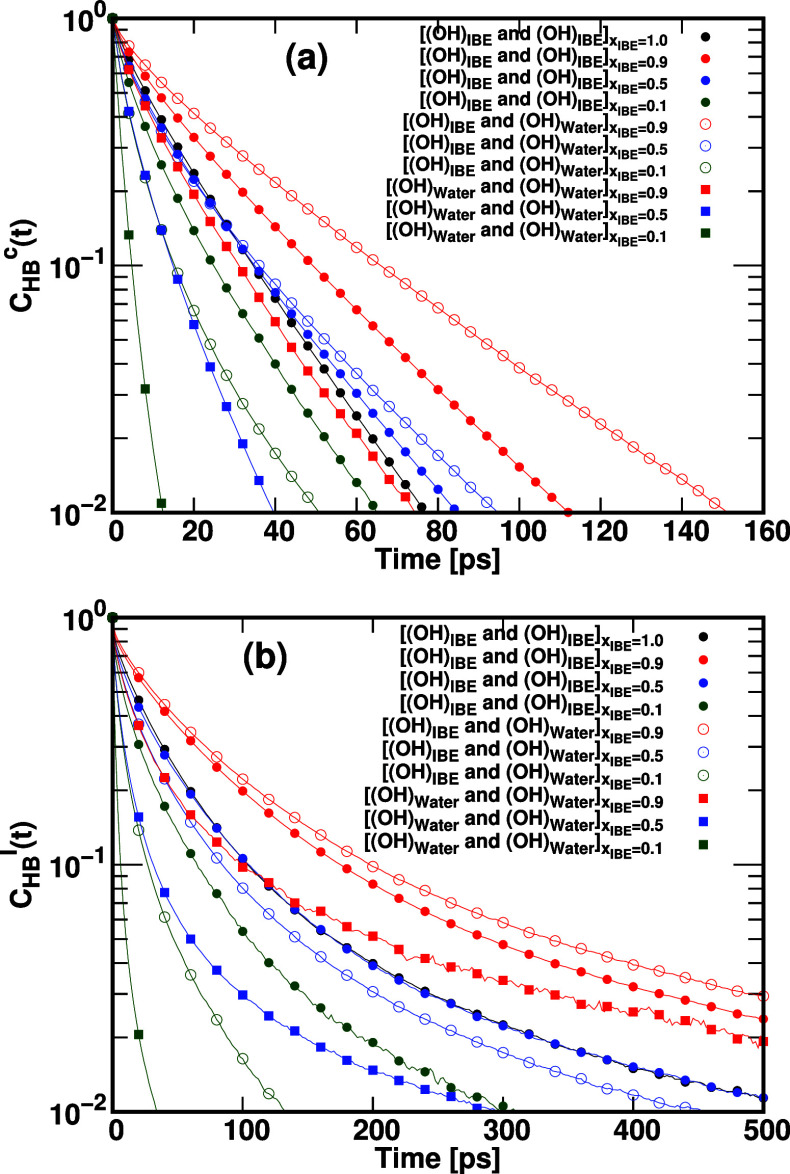
Hydrogen bond autocorrelation functions, specifically (a) the continuous *C*_HB_^c^(*t*) and (b) intermittent *C*_HB_^I^(*t*), for IBE and water molecules are illustrated. The investigated pairs include [(OH)_IBE_] and [(OH)_Water_].

**Table 3:** HB Relaxation Time τ_HB_ and Stretching Exponent β_HB_ for the IBE-Water Mixture

	continuous		intermittent		
H-bonding pair	τ_HB_^c^ [ps]	β_HB_^c^	τ_HB_^I^ [ps]	β_HB_^I^	*P*_HB_^I^
(IBE-IBE)_x_IBE_ = 1.0_	12.82	0.839	28.22	0.630	0.004
(IBE-IBE)_x_IBE_ = 0.9_	17.47	0.805	46.11	0.628	0.007
(IBE-IBE)_x_IBE_ = 0.5_	11.76	0.766	25.51	0.587	0.004
(IBE-IBE)_x_IBE_ = 0.1_	7.99	0.736	14.43	0.552	0.002
(IBE-Water)_x_IBE_ = 0.9_	23.20	0.797	51.98	0.630	0.007
(IBE-Water)_x_IBE_ = 0.5_	11.38	0.726	19.61	0.566	0.003
(IBE-Water)_x_IBE_ = 0.1_	4.76	0.705	5.93	0.538	0.001
(Water–Water)_x_IBE_ = 0.9_	10.48	0.773	18.57	0.495	0.007
(Water–Water)_x_IBE_ = 0.5_	4.87	0.746	6.13	0.490	0.003
(Water–Water)_x_IBE_ = 0.1_	1.72	0.815	1.52	0.564	0.001

The relaxation times of hydrogen bonds follow the order ([Water–Water]) < ([IBE-IBE]) < ([IBE-Water]). These results indicate that the continuous and intermittent hydrogen bond lifetimes (as shown in Figure 9 and Table 3) are significantly larger for ([IBE-Water]) and ([IBE-IBE]) compared to ([Water–Water]), mainly because the hydrogen bond dissociation barriers for ([IBE-Water]) and ([IBE-IBE]) are significantly larger than those of ([Water–Water])) (as observed in Figure 6). The continuous HB relaxation times (τ_HB_^c^) of (Water–Water) are 1.72, 4.87, and 10.48 ps in *x*_IBE_ = 0.1, 0.5, and 0.9, respectively. Therefore, the HB relaxation time increases with an increase in the mole fraction of IBE. In the mixtures with *x*_IBE_ = 0.5 and 0.9, HB relaxation times of ([Water–Water]) are significantly higher than those of *x*_IBE_ = 0.1, which supports the formation of highly ordered water clusters in *x*_IBE_ = 0.5 and 0.9. Consequently, the IBE plays a vital role in promoting the formation of more stable homogeneous clusters of water molecules in *x*_IBE_ = 0.5 and 0.9. The stretched exponential fitting of the hydrogen-bond correlation functions reveals β_HB_^c^ and β_HB_^I^ values below unity for all hydrogen-bonding pairs, reflecting the heterogeneous nature of H-bond dynamics in IBE–water mixtures. The degree of stretching becomes more pronounced (smaller β_HB_^c^ and β_HB_^I^) with increasing water content, indicating enhanced dynamic heterogeneity arising from the coexistence of IBE-rich and water-rich microdomains. In particular, β_HB_^I^ values obtained from the intermittent correlation functions are systematically lower than those from the continuous functions, signifying a broader distribution of relaxation times when H-bond breaking and reformation events are included. The minimum β_HB_^I^ values (0.5) around equimolar compositions suggest the strongest structural and dynamical heterogeneity due to competing IBE–IBE, IBE–water, and water–water interactions.

### 3.5 Viscoelastic Properties

Viscoelastic properties of the isotropic IBE-Water mixtures with *x*_IBE_ = 1.0, 0.9, 0.5, and 0.1 were calculated based on the stress–stress autocorrelation, which is defined as follows:

$$G(t)=\frac{1}{6k_{B}T}\left\{\left\langle \sigma_{xy}(t)\sigma_{xy}(0)\rangle +\langle \sigma_{yz}(t)\sigma_{yz}(0)\rangle +\langle \sigma_{zx}(t)\sigma_{zx}(0)\rangle +\frac{1}{4}[\langle N_{xy}(t)N_{xy}(0)\rangle +\langle N_{yz}(t)N_{yz}(0)\rangle +\langle N_{zx}(t)N_{zx}(0)\rangle \right]\right\}$$ 9

where the normal stress difference is defined as *N*_αβ_ = σ_αα_ – σ_ββ_.

In the case of *x*_IBE_ = 0.1, we observed phase separation between IBE and water. The observed phase separation shows a layer structure along the *z*-direction, as shown in Figure 1. Because the expression in eq 9 to obtain the relaxation modulus is valid only for an isotropic system, eq 9 should not be used for the system of *x*_IBE_ = 0.1. Instead, to investigate the linear viscoelastic property of the phase-separated system with a layered structure (Figure 1d), we calculated the quantities defined below. The in-plane viscoelastic property *G*_=_ of the mixture is calculated by

$$G_{=}(t)=\frac{V}{2k_{B}T}\left[\left\langle \sigma_{xy}(t)\sigma_{xy}(0)\rangle +\frac{1}{4}\langle N_{xy}(t)N_{xy}(0)\right\rangle \right]$$ 10

Note that ⟨σ_*xy*_(0)­σ_*xy*_(*t*)⟩ is statistically equivalent to $\frac{1}{4}\langle N_{xy}(t)N_{xy}(0)\rangle$ if the system has a rotational symmetry with respect to the *z*-axis. The viscoelastic property in the perpendicular direction to the phase-separated layer is evaluated by

$$G_{⊥}(t)=\frac{V}{2k_{B}T}\langle \sigma_{yz}(t)\sigma_{yz}(0)+\sigma_{zx}(t)\sigma_{zx}(0)\rangle$$ 11

The stress–stress autocorrelation function was evaluated by the multiple-tau method.^70^ The stress–stress autocorrelation function is shown in Figure 10 for each mixture. In the presence of water, the IBE-Water mixtures demonstrate noticeably slower relaxation dynamics relative to those of pure IBE. This deviation is primarily attributed to the strong intermolecular associations formed between the water and IBE molecules. In the mixtures with *x*_IBE_ = 0.1 and 0.5, the presence of stable, hydrogen-bond-mediated clusters between water and IBE further slows their relaxation dynamics compared to the pure IBE system (*x*_IBE_ = 1.0) and a mixture with *x*_IBE_ = 0.9, underscoring the influence of intermolecular interactions on the relaxation process. As the mole fraction of 2-isobutoxyethanol decreases, the plateau in the stress–stress autocorrelation function becomes more pronounced. Consequently, the binary IBE-Water mixture exhibits liquid-like behavior at higher IBE concentrations (*x*_IBE_ ≳ 0.9), whereas viscoelastic behavior dominates at lower mole fractions (*x*_IBE_ ≲ 0.5). Additionally, phase separation occurs at low IBE concentrations, notably, around *x*_IBE_ = 0.1. This behavior aligns with previous experimental findings. For instance, Perron et al. measured the thermodynamic properties of IBE-Water mixtures and reported the presence of two distinct phases at low IBE mole fractions.^1,2,5^

**Figure 10:**
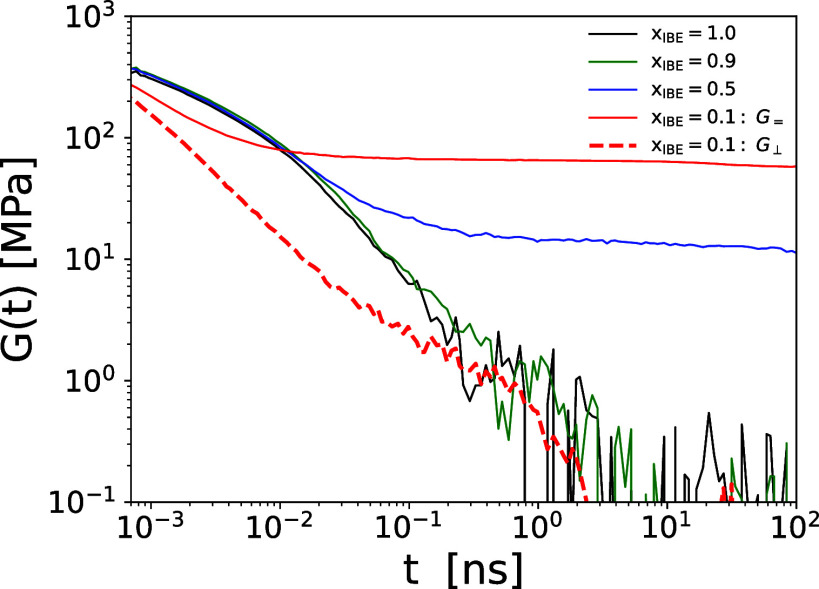
Relaxation moduli *G*(*t*) of the different types of mixtures of IBE-Water. *G*(*t*) is evaluated from the autocorrelation function of the stress tensor at equilibrium through eq 9. The in-plane viscoelastic property (*G*_=_), and the viscoelastic property in the perpendicular direction (*G*_⊥_) to the phase-separated layer of the mixture with *x*_IBE_ = 0.1 are shown by a solid red line and a dashed red line, respectively.

## 4 Conclusions

In this investigation, we have undertaken a comprehensive study to elucidate the role of the association between IBE-IBE, IBE-water, and water–water in the formation of inverse micromicellar and micromicellar structures in IBE-Water mixtures with various compositions. To gain in-depth insights, we employed all-atom molecular dynamics. Our analyses involved the calculation of equilibrium properties, including the dipole–dipole autocorrelation function ϕ­(*t*) and the dipole relaxation time τ_d_, diffusion coefficients, and stress–stress autocorrelation functions. Additionally, we explored the spatial arrangements of IBE around IBE, IBE around water, and water around water using radial distribution functions (RDFs). At *x*_IBE_ = 0.9, the dipole–dipole relaxation of IBE is significantly slowed relative to the pure system (*x*_IBE_ = 1.0), demonstrating that the introduction of even a small quantity of water strongly reduces molecular mobility. The effect persists at *x*_IBE_ = 0.5, where relaxation is still slower than that of pure IBE but moderately faster than that at *x*_IBE_ = 0.9. These trends are consistent with the interaction free energy profiles: at *x*_IBE_ = 0.9 and 0.5, IBE–Water interactions are stronger than IBE–IBE interactions, leading to slower dipole relaxation of IBE. Conversely, at *x*_IBE_ = 0.1, IBE–Water interactions are weaker than IBE–IBE; therefore, the dipole relaxation behavior of IBE is similar to that of *x*_IBE_ = 1.0. The dipole relaxation of IBE is slower in the *x*_IBE_ = 0.9 (Figure 7b) mixture in comparison to other mixtures because of the higher stability of the H-bond contact pair [[O_OH_]_IBE_] – [[H_OH_]_IBE_] of IBE-IBE and slower continuous and intermittent hydrogen bond correlation.

Furthermore, we examined the self-diffusion coefficient of IBE and water molecules to gain a comprehensive understanding of the dynamics of IBE in the presence of water, which revealed a distinct slowing in the diffusion behavior of IBE in IBE-Water mixtures. We also investigated the viscoelastic behavior of IBE-Water mixtures by computing stress–stress autocorrelation functions for each mixture. This observation provides strong evidence for the existence of clustering between the IBE and water molecules. To gain deeper insights, we conducted estimations of the radial distribution functions of the IBE around water, specifically focusing on [[O_OH_]_IBE_] – [[H_OH_]_IBE_], [[O_OH_]_Water_] – [[H_OH_]_Water_], [[O_OH_]_IBE_] – [[H_OH_]_Water_], and [[O_OH_]_IBE_] – [[O_OH_]_Water_] interactions (Figure 2). We have observed a significantly higher intensity of the first peak in the [[O_OH_]_IBE_] – [[H_OH_]_Water_] RDFs compared to those in [[O_OH_]_IBE_] – [[H_OH_]_IBE_] and [[O_OH_]_Water_] – [[H_OH_]_IBE_]. This compellingly indicates a much stronger association between IBE and water in comparison to IBE-IBE and Water–Water, as corroborated by the slower decay of hydrogen bond autocorrelation functions *C*_HB_^c^(*t*) and *C*_HB_^I^(*t*) for the IBE around water (Figure 9).

We have conducted a comprehensive analysis of the potential of the mean force between IBE and water molecules (Figure 4) and the cluster-formation fraction of terminal groups (Figure 5). We have assessed the interaction free energy between IBE-IBE, IBE-Water, and Water–Water using the potentials of mean force *W*(*r*). The [[O_OH_]_IBE_] – [[H_OH_]_IBE_], [[O_OH_]_Water_] – [[H_OH_]_Water_], and [[O_OH_]_IBE_] – [[H_OH_]_Water_] present a sharp contact minimum at approximately 0.2 nm, with free energy depths at these CMs significantly larger than the thermal energy. These findings provide evidence that the association between [[O_OH_]_Water_] – [[H_OH_]_IBE_] is more stable than that of [[O_OH_]_IBE_] – [[H_OH_]_IBE_], [[O_OH_]_Water_] – [[H_OH_]_Water_], as corroborated by hydrogen bond autocorrelation functions *C*_HB_^c^(*t*) and *C*_HB_^I^(*t*) for IBE around Water (Figure 9). Notably, we observed the presence of a contact minimum, solvent-shared minimum (SShM), and solvent-separated minimum (SSM) for [[O_OH_]_Water_] – [[H_OH_]_IBE_], [[O_OH_]_IBE_] – [[H_OH_]_IBE_], and [[O_OH_]_Water_] – [[H_OH_]_Water_]. We also explored the cluster-formation-fraction *f*_cluster_^(X)^(*s*) using two criteria: (i) the distance criterion and (ii) the potential energy criterion (|*W*(*r*_CM_)| > *k*_B_ *T*). The clustering behavior observed in IBE-Water mixtures highlights the competition between the hydrophobic association of IBE molecules and the hydrogen-bond-driven aggregation of water molecules. At low IBE mole fraction (*x*_IBE_ = 0.1), large IBE clusters are formed (Figure 6a). At intermediate IBE fraction (*x*_IBE_ = 0.5), the detection of large water clusters (sizes 2–31) (Figure 6b) suggests that the hydrophilic headgroup of IBE is strongly associated with these water clusters and the hydrophobic tail is pointed away from the water clusters. When *x*_IBE_ = 0.5 or 0.9, heterogeneous clusters with water aggregates surrounded by IBE molecules are shown in Figure 6c, resembling inverse micelle structures that are known in other amphiphilic systems. Analogous behavior in IBE–Water systems supports the plausibility of inverse micellar arrangements under hydrophobic-rich compositions.^18^ At a low IBE fraction (*x*_IBE_ = 0.1), we also observed the heterogeneous clusters (IBE)_p_(H_2_O)_*q*_ with *p* = 3–31 and *q* = 1 (Figure 5d), where water molecules localize at the IBE cluster periphery in which hydrophilic hydroxy groups of IBE are located at the outer of the IBE cluster and strongly associated with the water molecules while hydrophobic tail of IBE molecules are present in the interior of the IBE cluster and these IBE tails are strongly associated with each other and facilitate the formation of the micromiceller structure. The formation of micromicellar structure at low mole fraction of IBE results in phase separation in the IBE-Water mixture. Our investigations are supported by previous research.^5,18^ Thus, the balance between hydrophobic tail length, headgroup solvophilicity, and Water–Water hydrogen bonding appears central in determining whether micellar, inverse micellar, or heterogeneous cluster structures dominate. To recapitulate, our investigation probed the impact of H-bonds both on the association of IBE-IBE, IBE-Water, and Water–Water, and on the dynamic behavior of IBE and water molecules. The presence of a high intensity first RDF peak at 0.2 nm between [[O_OH_]_Water_] – [[H_OH_]_IBE_] confirms the formation of a strong hydrogen bond between the IBE and water molecules (Figure 2). The robust hydrogen-bond interactions observed in [[O_OH_]_Water_] – [[H_OH_]_IBE_] facilitated the formation of substantial, stable heterogeneous clusters between IBE and water with sizes greater than 2. These heterogeneous clusters are connected through IBE molecules via a hydrogen bond and facilitate the formation of a network structure. We have validated the presence of highly stable heterogeneous clusters formed by robust hydrogen-bond interactions among the IBE and water molecules. The compositional dependence of the stress–stress autocorrelation function provides insight into the underlying structural and dynamic changes in the 2-isobutoxyethanol (IBE)–water binary mixture. A systematic increase in the plateau region is observed as the mole fraction of IBE decreases due to the formation of inverse micromicellar and micromicellar structures. At higher IBE concentrations (*x*_IBE_ ≳ 0.9), the system predominantly exhibits viscous liquid-like dynamics, because of the absence of inverse micromicellar and a micromicellar structures. In contrast, at lower mole fractions (*x*_IBE_^*^ ≲ *x*_IBE_ ≲ 0.5, *x*_IBE_^*^ is a small value at least greater than 0.01 at which we have confirmed by our simulations that no phase separation occurs, but the result is not shown here), the increased prominence of viscoelastic behavior can be attributed to the existence of micelle structures in *x*_IBE_ = 0.1 (Figures 5d and 6d, and Video S1) and inverse micelle in *x*_IBE_ = 0.5 (Figures 5c, 6c, and[Fig fig11]; Video S2). At a very low IBE concentration region (*x*_IBE_ ≲ *x*_IBE_^*^), the system shows a single homogeneous phase and behaves as a viscous liquid. The phase separation observed at *x*_IBE_ = 0.1 indicates that these structural changes extend to the macroscopic scale, likely arising from the limited miscibility of IBE in water at low concentrations. These results are in good agreement with previous experimental observations.^1–5^ In particular, Perron et al.^2^ measured the thermodynamic properties of IBE-Water mixtures and confirmed the existence of two distinct liquid phases at low IBE mole fractions, further supporting the interpretation that molecular-level interactions govern the observed viscoelastic and phase behaviors. A subsequent study will systematically examine how shear deformation, applied at different strain rates, affects the phase behavior of IBE–water mixtures.

**Figure 11:**
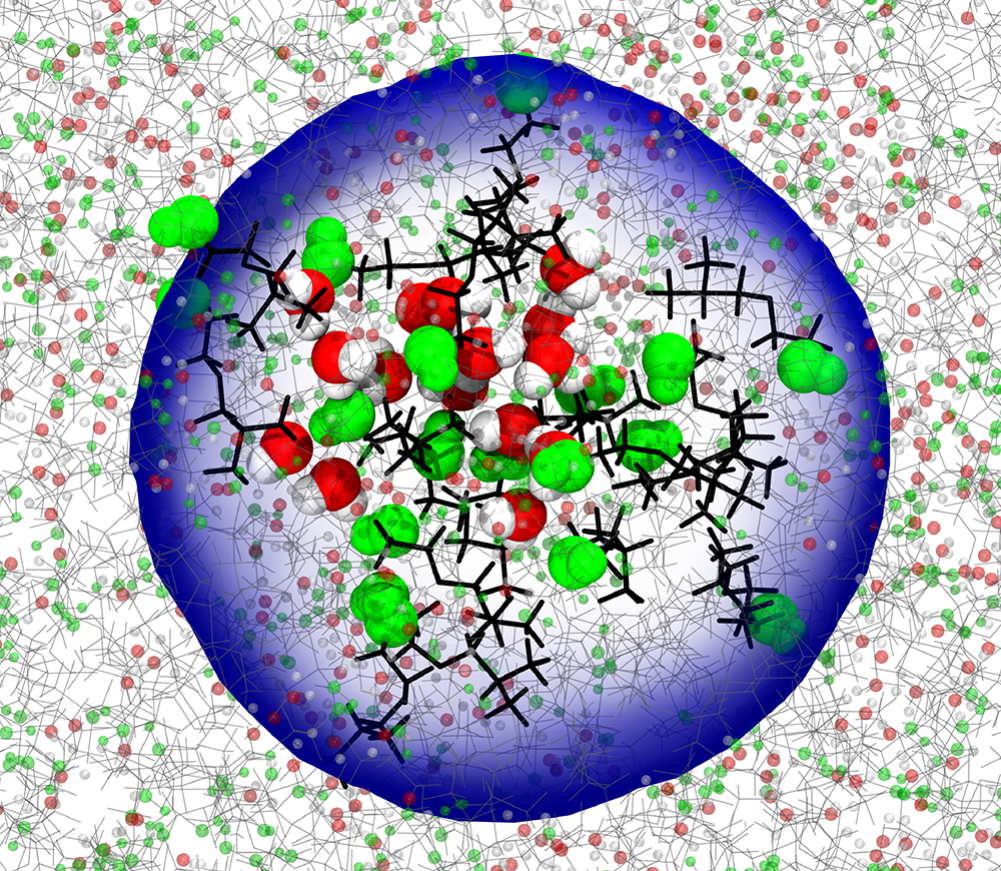
Cluster of water molecules enclosed by IBE molecules via a H-bond, like an inverse micromicellar structure in the *x*_IBE_ = 0.5 mixture. The carbon, hydrogen, and oxygen atoms are shown in cyan, white, and red colors, respectively. The hydroxy groups of IBE are shown by a green-colored sphere.

## Supplementary Material







## Notes

Initial structures of IBE-Water mixtures (Figure S1); radial distribution functions between the backbone of IBE molecules (Figure S2); rotational correlation functions as a function of time (Figure S3); intermittent hydrogen bond autocorrelation function *C*_HB_^I^(*t*) (Figure S4); running coordination numbers in the first and second coordination shells of IBE around IBE, IBE around water, water around IBE, and water around water in IBE and water mixtures (Table S1); hydrogen bond (HB) relaxation time τ_HB_ and stretching exponent β_HB_(Table S2); Figures S1 to S4; and Table S1 for reference and additional information (PDF)
Existence of micromicelle structures in *x*_IBE_ = 0.1 (MP4)
Existence of inverse micro micelle structures in *x*_IBE_ = 0.5 (MP4)
