# Supplementary material for: Micelle and Inverse Micelle Structure Driven Viscoelasticity and Phase Separation in 2‑Isobutoxyethanol–Water Mixtures: Insights from All-Atom Simulations
Source: Precis Chem. 2025 Dec 8;4(3):240–52. doi: 10.1021/prechem.5c00104 (PMC13014340; doi:10.1021/prechem.5c00104)
Supplement: Supplementary file 1 [file pc5c00104_si_001.pdf]

Supporting Information: Micelle and Inverse Micelle Structure Driven Viscoelasticity and Phase Separation in 2-Isobutoxyethanol–Water Mixtures: Insights from All-Atom Simulations

Mayank Dixit,*,† Kenji Sugase,*,‡ and Takashi Taniguchi*,†

†*Graduate School of Engineering, Kyoto University, Nishikyo-ku, Kyoto 615-8510, Japan*

‡*Graduate School of Agriculture, Kyoto University, Department of Applied Life Sciences Biopolymer Chemistry (concurrently Applied Structural Biology) N346, Faculty of Agriculture Building, Kitashirakawa Oiwake-cho, Sakyo-ku, Kyoto 606-8502, Japan*

E-mail: dixit@cheme.kyoto-u.ac.jp; sugase.kenji.8c@kyoto-u.ac.jp; taniguch@cheme.kyoto-u.ac.jp

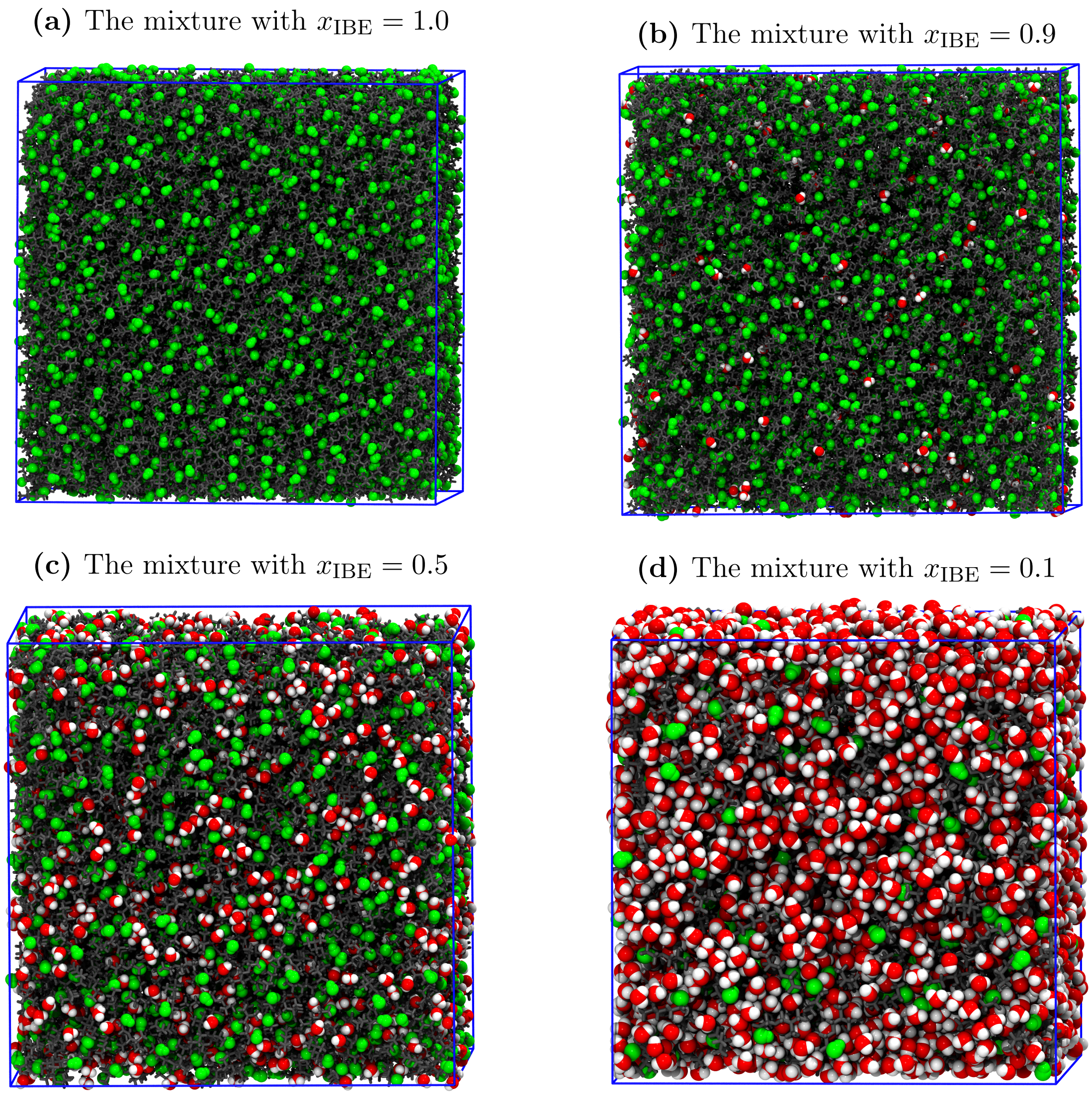

(a) The mixture with $x_{\mathrm{IBE}} = 1.0$

(b) The mixture with $x_{\mathrm{IBE}} = 0.9$

(c) The mixture with $x_{\mathrm{IBE}} = 0.5$

(d) The mixture with $x_{\mathrm{IBE}} = 0.1$

Figure S1: The initial structure of isobutoxyethanol (2-isobutoxyethanol ($(CH_3)_3CH$-OCH_2CH_2-OH), IBE)/water mixtures with $x_{\mathrm{IBE}} = 1.0$ (a), $x_{\mathrm{IBE}} = 0.9$ (b), $x_{\mathrm{IBE}} = 0.5$ (c),$x_{\mathrm{IBE}} = 0.1$ (d). The carbon, hydrogen, and oxygen atoms are shown by cyan, white, and red colors, respectively.

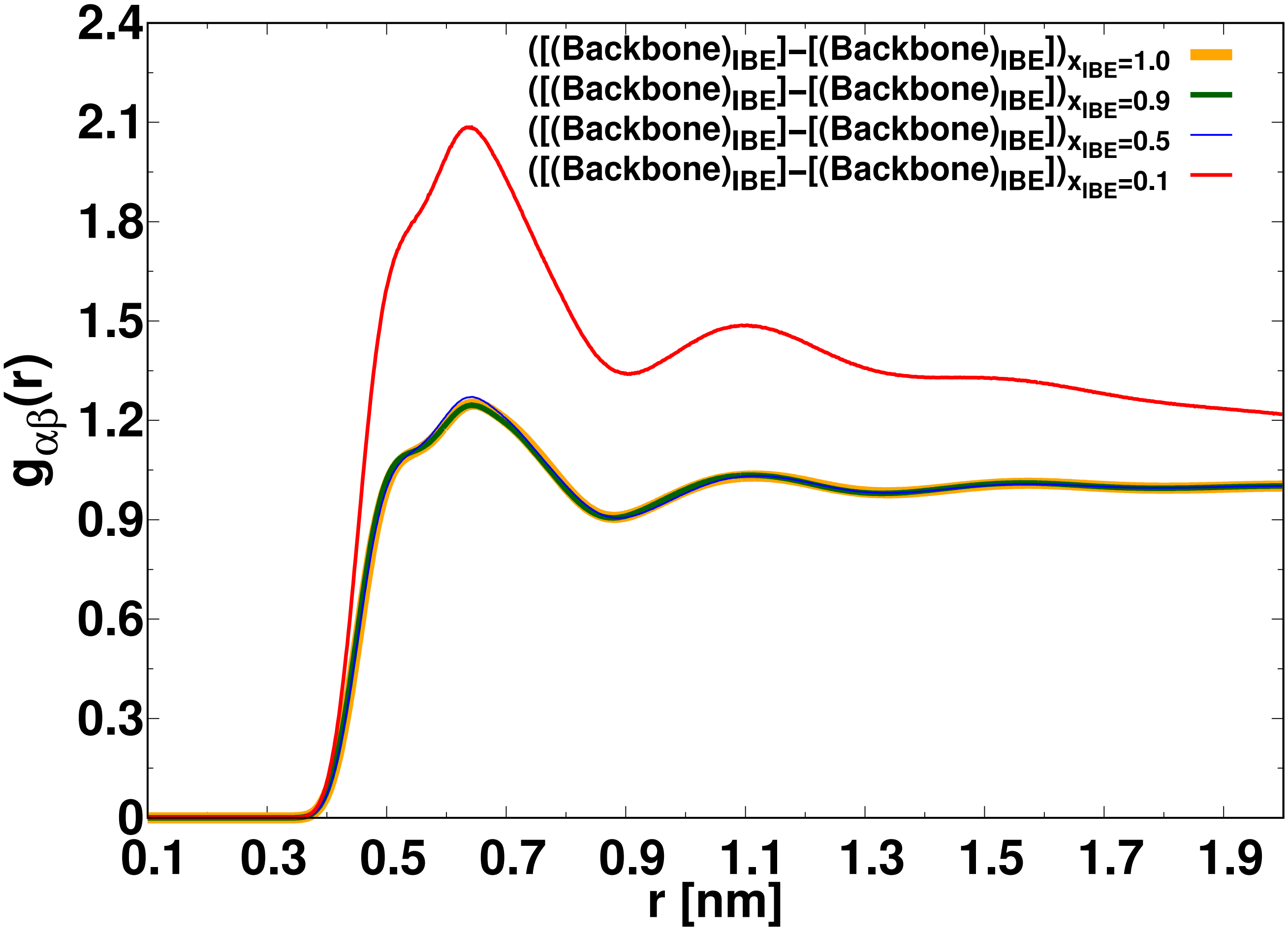

Figure S2: The radial distribution functions (RDFs) between the backbone of IBE molecules in the IBE-water mixtures.

(a) The rotational relaxation vs time for IBE, and Water

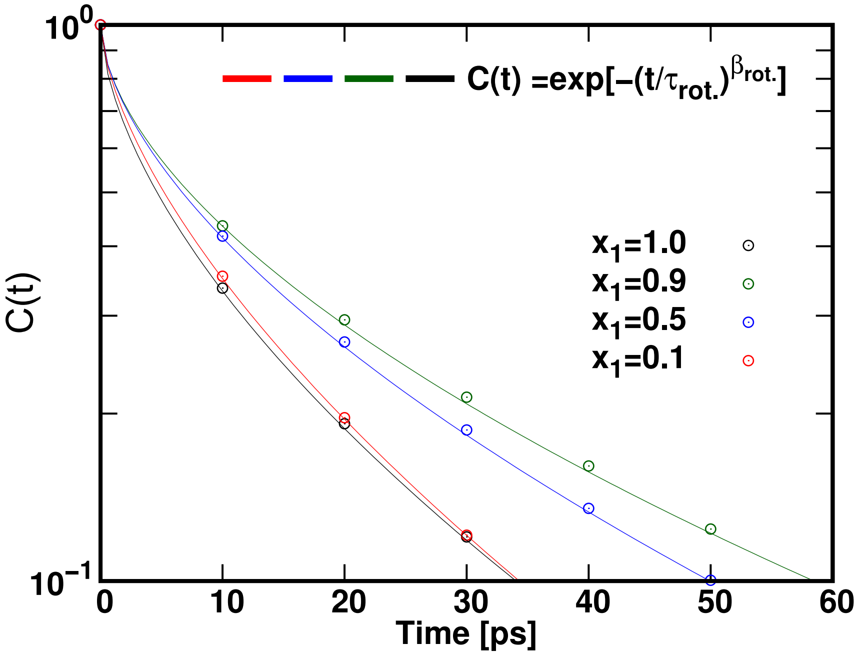

(b) Average values of rotational relaxation time (τ_{rot}) for IBE and water.

System	Relaxation time τ_{rot} [ps]	
	$\beta_{\mathrm{rot}} = 1$	$\beta_{\mathrm{rot}} \neq 1$
$x_1 = 1.0$	10.28	8.51 ($\beta_{\mathrm{rot}} = 0.60$)
$x_1 = 0.9$	17.01	13.72 ($\beta_{\mathrm{rot}} = 0.57$)
$x_1 = 0.5$	15.00	12.36 ($\beta_{\mathrm{rot}} = 0.59$)
$x_1 = 0.1$	10.83	9.26 ($\beta_{\mathrm{rot}} = 0.63$)

Figure S3: The rotational correlation functions as a function of time are depicted for IBE and water. Panel (a) displays the fits to the stretched exponential function, $C(t) = \exp[-(t/\tau_{\mathrm{rot}})^{\beta_{\mathrm{rot.}}}]$, represented by solid circles. Panel (b) presents the corresponding rotational relaxation times, denoted as τ_{rot}, computed by fitting the end-to-end vector autocorrelation functions, $C(t)$, to both the simple exponential and stretched exponential functions.

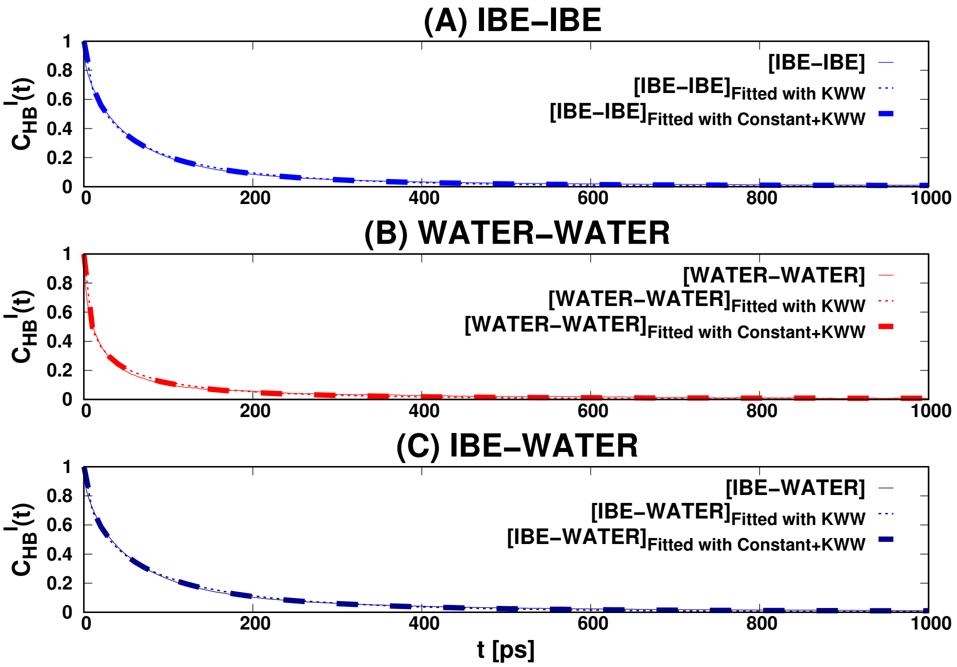

Figure S4: The hydrogen bond correlation functions, intermittent $C^{\mathrm{I}}_{\mathrm{HB}}(t)$, for IBE and water molecules are illustrated. The pairs investigated include ([[OH]$_{\mathrm{IBE}}$], ([[OH]$_{\mathrm{water}}$].

Table S1: Running coordination numbers in the first and second coordination shells of IBE around IBE, IBE around Water, Water around IBE, and Water around Water in IBE and water mixtures.

Mixtures	IBE around IBE		IBE around Water		Water around IBE		Water around Water	
	First Shell	Second Shell	First Shell	Second Shell	First Shell	Second Shell	First Shell	Second Shell
$x_1 = 1.0$	1.46	3.90	-	-	-	-	-	-
$x_1 = 0.9$	1.39	4.09	2.32	4.11	0.26	0.47	0.41	0.52
$x_1 = 0.5$	0.88	3.75	1.38	3.87	1.38	3.95	2.42	5.12
$x_1 = 0.1$	0.53	2.57	0.27	1.06	2.42	9.75	5.76	19.36

Table S2: The hydrogen bond (HB) relaxation time τ_{HB} and stretching exponent β_{HB} of $[[\mathrm{O_{OH}}]_{\mathrm{IBE}}]$-$[[\mathrm{H_{OH}}]_{\mathrm{Water}}]$, $[[\mathrm{O_{OH}}]_{\mathrm{IBE}}]$-$[[\mathrm{H_{OH}}]_{\mathrm{IBE}}]$ and $[[\mathrm{O_{OH}}]_{\mathrm{Water}}]$-$[[\mathrm{H_{OH}}]_{\mathrm{IBE}}]$ in IBE-water mixture with $x_{\mathrm{IBE}} = 0.9$.

H-bonding pair	HB relaxation time (τ_{HB})/[ps]		Stretching exponent (β_{HB})		Constant ($P'^{\mathrm{I}}_{\mathrm{HB}}$)	χ^2	
	($\tau'^{\mathrm{I}}_{\mathrm{HB}}$)	($\tau^{\mathrm{I}}_{\mathrm{HB}}$)	($\beta'^{\mathrm{I}}_{\mathrm{HB}}$)	($\beta^{\mathrm{I}}_{\mathrm{HB}}$)	($P^{\mathrm{I}}_{\mathrm{HB}}$)	$(\chi'^{\mathrm{I}}_{\mathrm{HB}})^2$	$(\chi^{\mathrm{I}}_{\mathrm{HB}})^2$
IBE – IBE	47.84±0.20	46.11±0.10	0.6030±0.0021	0.6278±0.0013	0.0073±0.0001	4.86×10^{-5}	1.41×10^{-5}
Water – Water	19.37±0.18	18.57±0.12	0.4605±0.0024	0.4952±0.0021	0.0074±0.0002	6.67×10^{-5}	3.33×10^{-5}
IBE – Water	54.86±0.22	51.98±0.12	0.6012±0.0020	0.6277±0.0013	0.0078±0.0001	5.24×10^{-5}	1.60×10^{-5}
